# Dispersion of TiO_2_ nanoparticles improves burn wound healing and tissue regeneration through specific interaction with blood serum proteins

**DOI:** 10.1038/s41598-017-15792-w

**Published:** 2017-11-13

**Authors:** Gulaim A. Seisenbaeva, Karin Fromell, Vasiliy V. Vinogradov, Aleksey N. Terekhov, Andrey V. Pakhomov, Bo Nilsson, Kristina Nilsson Ekdahl, Vladimir V. Vinogradov, Vadim G. Kessler

**Affiliations:** 1 Department of Chemistry and Biotechnology, BioCenter, Swedish University of Agricultural Sciences, Box 7015, SE-750 07 Uppsala, Sweden; 2 Department of Immunology, Genetics and Pathology, Rudbeck Laboratory C5:3, Uppsala University, SE-751 85 Uppsala, Sweden; 3 Laboratory of Solution Chemistry of Advanced Materials and Technologies, ITMO University, Kronverksky Pr. 49, St, Petersburg, 197101 Russian Federation; 4 Ivanovo State Medical Academy, Sheremetevskiy prosp. 8, Ivanovo, 153012 Russian Federation; 5 Linnæus Centre for Biomaterials Chemistry, Linnæus University, SE-391 82 Kalmar, Sweden

## Abstract

Burn wounds are one of the most important causes of mortality and especially morbidity around the world. Burn wound healing and skin tissue regeneration remain thus one of the most important challenges facing the mankind. In the present study we have addressed this challenge, applying a solution-stabilized dispersion TiO_2_ nanoparticles, hypothesizing that their ability to adsorb proteins will render them a strong capacity in inducing body fluid coagulation and create a protective hybrid material coating. The *in vitro* study of interaction between human blood and titania resulted at enhanced TiO_2_ concentrations in formation of rather dense gel composite materials and even at lower content revealed specific adsorption pattern initiating the cascade response, promising to facilitate the regrowth of the skin. The subsequent *in vivo* study of the healing of burn wounds in rats demonstrated formation of a strongly adherent crust of a nanocomposite, preventing infection and inflammation with quicker reduction of wound area compared to untreated control. The most important result in applying the TiO_2_ dispersion was the apparently improved regeneration of damaged tissues with appreciable decrease in scar formation and skin color anomalies.

## Introduction

Accelerated and less painful healing of wounds caused by burn or mechanical injuries and skin and muscle tissue engineering for decreased scar formation and minimization of permanent damage belong to most prominent challenges in modern surgery^1^. Application of nanostructured materials for improved tissue regeneration has become a well-developed and accepted practice in the application of metal bone implants, where a thin layer of nanostructured titanium dioxide is deposited on the top of the implant surface^2,3^. Nano titania is rapidly getting coated with proteins when immersed into the biological fluids due to its well-recognized ability to adsorb and coordinate proteins^4^ and phospholipids^5^ on its surface. Adsorption of phospholipids can be considered as one of the factors guiding the attachment of cells and grafting on the growing tissue on an implant^6^. In the domain of skin regeneration a strong effort so far has been set on application of stem cells. They have been applied in different approaches, in particular, including sprays^7^. Use of nanomaterials for wound treatment and skin repair has also been intensively investigated ranging from silicone based artificial skin layers^8^, to the development of new materials for wound dressing with delayed and prolonged release of medicines^9,10^ and even to direct application of nanoparticle dispersions either possessing themselves antibacterial effects, such as silver and gold nanoparticles^11–13^, or loaded with painkillers and antibiotics. The latter have been realized with metal oxide nanoparticles which the FDA approved for intravenous application, namely with Al_2_O_3_^14^ or with Fe_3_O_4_^15^ as carriers. The use of medicine-loaded sol-gel alumina and iron oxide resulted in appreciable reduction of the scar tissue sizes with wound healing times not principally different, however, from those when only a solution of the selected medicines was applied directly to the wound^14,15^. Considerable improvement in the size and structure of the scar could be observed when anti-inflammatory natural medicine curcumin loaded siloxane gels^16^, while using silver nanoparticle-graphene-polymer nanocomposites an appreciable acceleration of wound healing was achieved^17^. Nano TiO_2_ has also drawn a considerable attention in the recent years. In the view of broad application of nano titania a lot of effort were set on, in the first hand, investigation of its potential toxicity in different forms^18^. It has been demonstrated in numerous studies reviewed, analyzed and reproduced in^19^ that in the dark no or negligible toxicity could be associated with essentially any form of nano titania. The apparent toxicity, in particular DNA damage, could be observed for both human cells^19^ and for microorganisms, such as, for example, micro algae^20^ in the presence of highly crystalline larger (over 25 nm and in most apparent cases – about 100 nm^20^) particles, obtained by spray pyrolysis, subjected to irradiation by UV and visible^20^ and in some cases even IR light^21^. This effect was unequivocally related to the photocatalytic properties of the applied titania and was more pronounced for the more catalytically active anatase phase^20^. The application of nano TiO_2_ in wound healing was also very much focused on the exploitation of the photocatalytic effect for production of reactive oxygen species to target bacteria in wound infections^21,22^. To enhance the bactericidal action of titania via photochemical effects, quite a number of applications have been developed for grafting titania onto either fabrics^23,24^ or porous (hydrophilic) polymer nano composites^25,26^. In some cases such composites were used, however, primarily for controlled drug delivery to the wounds^9,10^.

In the present work we have chosen a completely different approach in use of nano titania for wound healing. The material applied here was a dispersion of small (less than 10 nm) anatase particles produced by sol-gel method in solution. The colloid was stabilized by grafting of (protonated) antioxidant ligand triethanolamine on the surface of the particles, making them positively charged in the originally applied media and photochemically inactive^27^. The small anatase particles stabilized by antioxidant ligands have been demonstrated to be biocompatible for both human cells^28^ and for bacteria^27^ and micro algae^29^. The applied dispersion did not contain any additional bioactive substances or medicines. The aim was set to investigate possible effects originating solely via surface interactions (adsorption) of the nano titania, possessing large active surface area, with body fluids.

## Results and Discussion

Nanoparticles of titania, independently of their phase composition and in spite of their broadly recognized adsorbent properties towards biomolecules, are commonly considered as inert in relation to living systems^18^. Titania as anatase powder is broadly used in food and hygiene industry, referred to as E171 food colorant. Commercially available Degussa P25 titania nano powder is considered as broadly accepted negative standard in the *in vitro* acute toxicity studies^18^. The dispersion of sol-gel produced anatase nanoparticles stabilized by charging via surface complexation with triethanolammonium ligands applied in this work has been characterized in several earlier publications and was proved to be biocompatible in contact with both human and plant cells up to rather high concentrations reaching 100 μg/mL^30,31^. Recent investigation of the Degussa P25 nanoparticles at very low concentration of below 50 ng/mL has demonstrated them to be capable to induce activation of the contact system eliciting thromboinflammation^32^.

**Figure 1:**
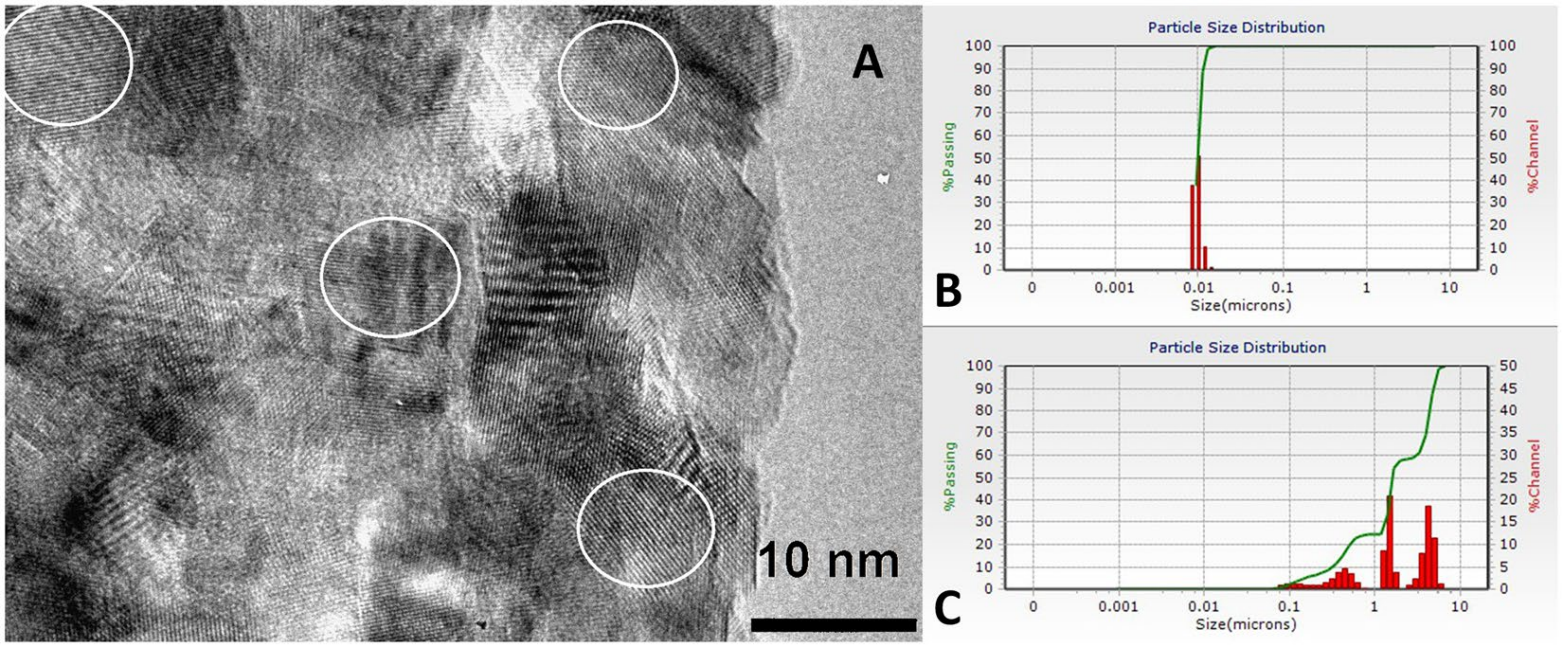
TEM image of the dried dispersion of the applied TiO_2_ nanoparticles produced using modified methodology from ref.^18^ (**A**). Hydrodynamic size of the particles in water (**B**) and in isotonic salt solution ((**C**), 0.5 ml of dispersion diluted by 10 ml isotonic NaCl), both by DLS.

This latter observation was considered to be associated with potential health risk from titania nanoparticles if they emerge in the body fluids. On the contrary, we hypothesized that a dispersion of TiO_2_ nanoparticles should be applied on the skin to cause enhanced blood coagulation, which is an important first step in initialization of the wound healing processes. The nanoparticles applied in this work were produced by hydrolytic route from titanium ethoxide modified by triethanolamine ligands, following the procedure adopted from^27^ with some minor adjustments (Please, see the experimental part and Supplementary). They belong to the anatase phase as is clearly indicated by the distances between fringes for the aligned {101} planes of 0.354 nm in the high resolution TEM images (see Fig. 1A and Figs S1, S3 and S4). The size of the particles is rather uniform and is well in agreement with the observed hydrodynamic size in both the initial alcohol-based dispersion and in the dispersion obtained by its 10 times dilution by de-ionized water (Figs 1B and S2). In contrast, the dilution by 0.9 wt% NaCl (physiological solution), while not leading to precipitation is associated with extended aggregation and apparent increase in the hydrodynamic size of the produced aggregates with distribution between about 100 nm and several micrometers (Fig. 1C).

**Figure 2:**
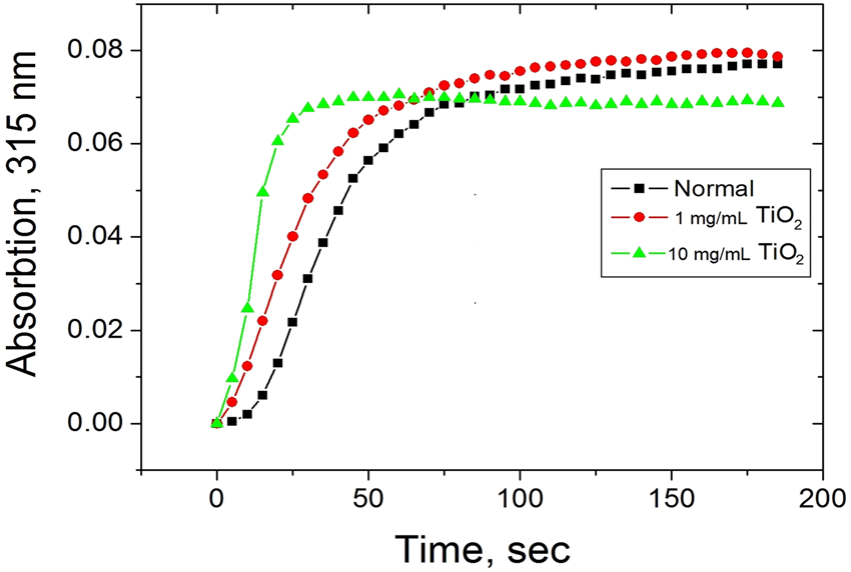
Accelerated blood clotting on addition of sol-gel titania, represented by average values of 3 measurements for each of the curves.

Spectrophotometric measurements of the clot formation at quite enhanced final TiO_2_ concentrations in human blood plasma (1 mg/mL and 10 mg/mL respectively) showed that it was strongly accelerated compared to normal clotting in air. The process was completed in just over 30 s at 1 mg/mL and in about 10 s for 10 mg/mL compared to over 1 min with untreated blood serum (see Fig. 2).

**Figure 3:**
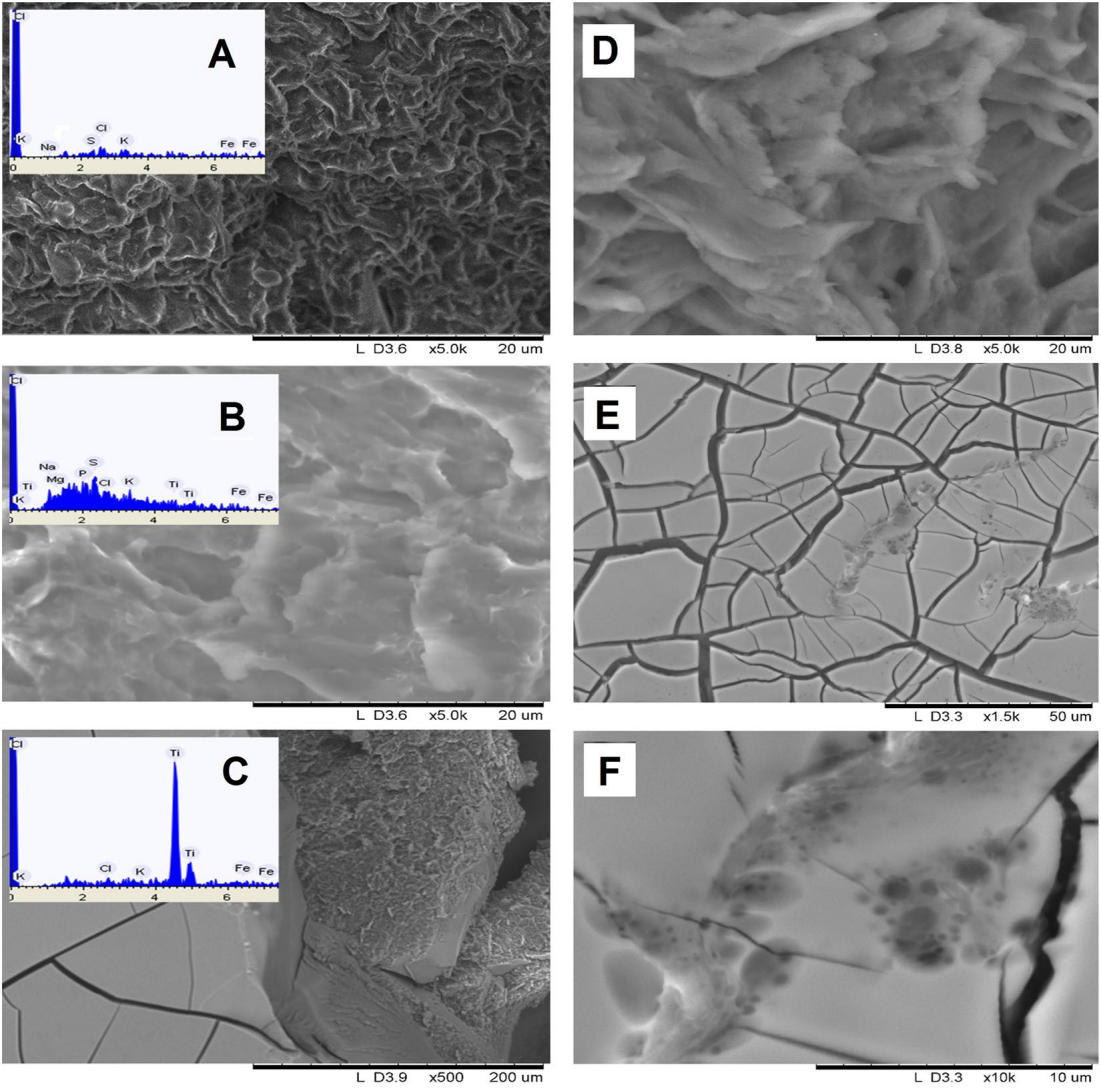
SEM images illustrating interaction of nano titania dispersion with human blood and skin samples: Natural blood clot and its EDS analysis (**A**), enlarged structure of the inner part of the blood clot forming on interaction with the TiO_2_ dispersion and its EDS analysis (**B**), TiO2 crust on the surface of a treated blood clot and its EDS analysis (**C**). The integrations of EDS spectra are presented in Table S1 (Supplementary). Untreated skin sample (**D**) coating on the surface of skin sample (**E**), enlarged view of the titania film on a skin sample (**F**).

In order to bring insight into the interaction of blood with the applied titania dispersion, we produced clots by addition of a droplet (0.02 mL) of the dispersion (or PBS solution for reference) to 0.10 mL of whole blood drop placed onto an optical glass slide. Quick clotting in case of TiO_2_ dispersion led to formation of a composite in a form of dark brown, almost black, brittle solid. The SEM analysis revealed formation of a dense solid TiO_2_ film on the outer surface of solidified droplet with thickness of 30–40 μm, covering a complex structure of iron-rich protein composite with only 3–4 wt% content of titania as calculated from EDS analysis (see Fig. 3B,C and Supplementary Fig. S3, and Table S1).

The structure of the protein clot is apparently different for pure blood or the blood with added PBS solution on one hand, and the material produced on interaction with titania dispersion on the other hand: the microstructure in the latter case is larger and more smooth, indicating stronger interactions within the clot. It indicates that the addition of titania is resulting in stronger interactions within the forming solid. We have applied rather high concentration of titania aiming to produce a dense nanocomposite material with perspective to form such composite coatings on the wounds as protective patches instead of using polymer patches as recommended earlier^9,23–26^. Interaction of the dispersion with freshly removed human epidermis (outer skin layer) was also investigated, showing that the complex skin structure becomes coated with a uniform dense layer of solid titania film (Fig. 3D–F) with thickness dependent of the concentration and amount of added dispersion, but typically thicker than 10 μm. The thickness of a film obtained by single deposition is apparently quite high, which leads to formation of a uniform system of surface cracks originating from gel densification on the evaporation of solvent. The coating has apparently good adhesion to the skin surface and is not removed mechanically when dry. Washing off with mechanical brushing in a water flow removes major part of it with residues remaining persistently in the skin micro folds. These features in combination indicated that treatment of the wounds with the applied concentrated titania dispersion were going to create on their surface quite dense mechanically tough and strongly adhering hybrid coatings. Such coatings could be potentially capable to serve as protective patches on their own, eliminating the need to cover a wound with some additional protective material/bandage.

**Figure 4:**
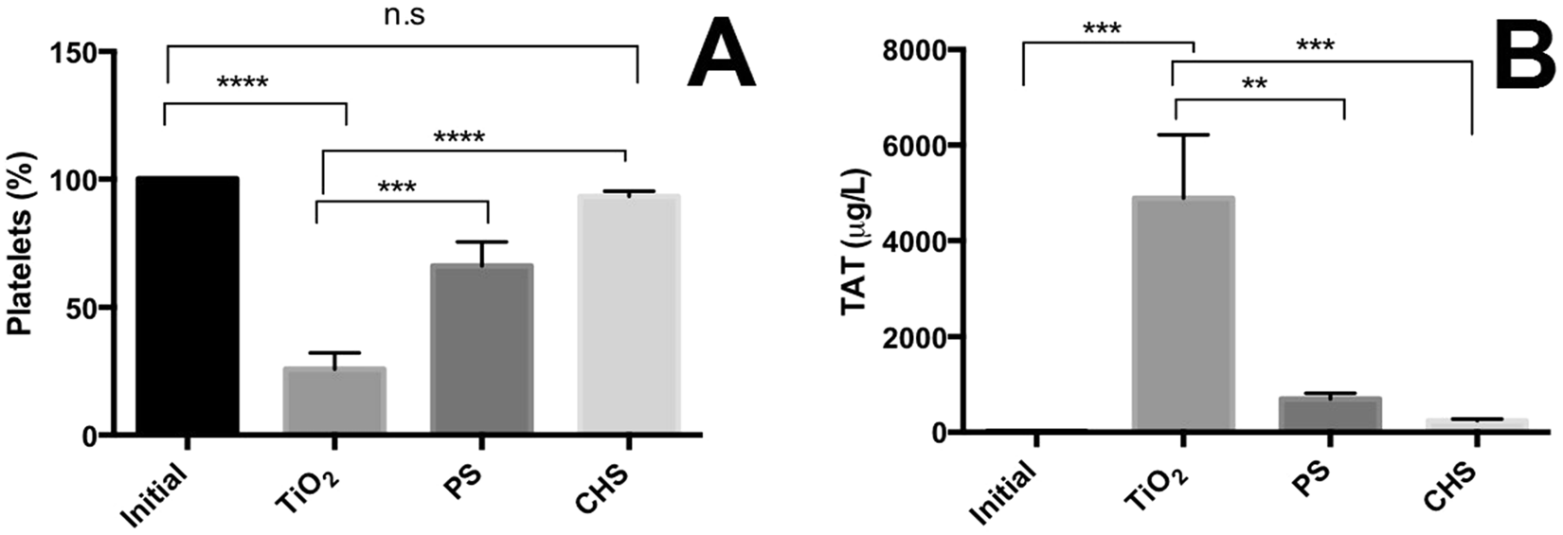
(**A**) Remaining platelets in whole blood after contact with the titania (TiO_2_), polystyrene (PS) and Corline heparin (CHS) surfaces for 60 min at 37 °C. Data represent mean ± SEM (n = 4). (**B**) Formation of Thrombin-Antithrombin complex in contact with the titania (TiO_2_), polystyrene (PS) and Corline heparin (CHS) surfaces. Data represent mean ± SEM (n = 4). Significance was determined by One Way ANOVA analysis using Dunnett’s multiple comparison test. Significant differences are indicated as **p < 0.01; ***p < 0.001; ****p < 0.0001; n.s = non-significant. Results were considered statistically significant for p < 0.05.

Platelet activation was measured as the reduction in platelet numbers in the blood after incubation with the TiO_2_ nanoparticle-coated surface (TiO_2_), polystyrene surface (PS) and Corline heparin surfaces (CHS) compared to the initial blood samples (i.e., not exposed to the chambers). The TiO_2_ surface induced a clear reduction in platelets as only 25.8 ± 6.4% (mean ± SEM) of the platelets remained in the blood after the incubation, while the blood incubated with the plain PS surface still had 66 ± 9.4% of the platelets and the in the blood from the CHS surface more than 90% (93.2 ± 2.1%) of the platelets were remaining after the same incubation time (See Fig. 4A).

Activation of the coagulation system was further analyzed by measuring the generation of TAT complexes after blood exposure to the TiO_2_ nanoparticle containing surface, the PS surface and the CHS surface. As expected the TiO_2_-nanoparticle coated surface resulted in a large increase in TAT levels compared to the PS surface that just showed a small increase in TAT levels, and the CHS control surface that gave no significant rise in TAT concentration compared to the initial blood samples (See Fig. 4B).

The intrinsic pathway of coagulation is triggered when FXII come in contact with foreign materials, which initiates the clot formation and thereby also the wound healing process. Hence, it would be of importance to see if the TiO_2_ nanoparticle-coated surfaces induce this type of contact activation. In a previous publication we investigated protein adsorption and contact system activation induced by TiO_2_ nanoparticles incubated in human EDTA-plasma and whole blood without anticoagulantia, respectively^32^. The formed protein corona was abundant in most contact activation proteins; five out of the ten protein identified with highest score identified by MALDI-TOF belonged to the contact system. High amounts of contact system activation complexes were generated reflecting this binding^32^. In the present study, the generation of FXIIa-AT and FXIIa-C1INH complexes was measured in the blood after incubation with the TiO_2_ coated surface, with and without the FXII-specific inhibitor CTI. The result showed that both FXIIa-AT and FXIIa-C1INH complexes were formed in the plasma after contact with the TiO_2_ nanoparticle coated surface, but the addition of CTI inhibited the formation of these complexes with ca. 80%, thus confirming an FXIIa-dependent complex formation (data not shown).

**Figure 5:**
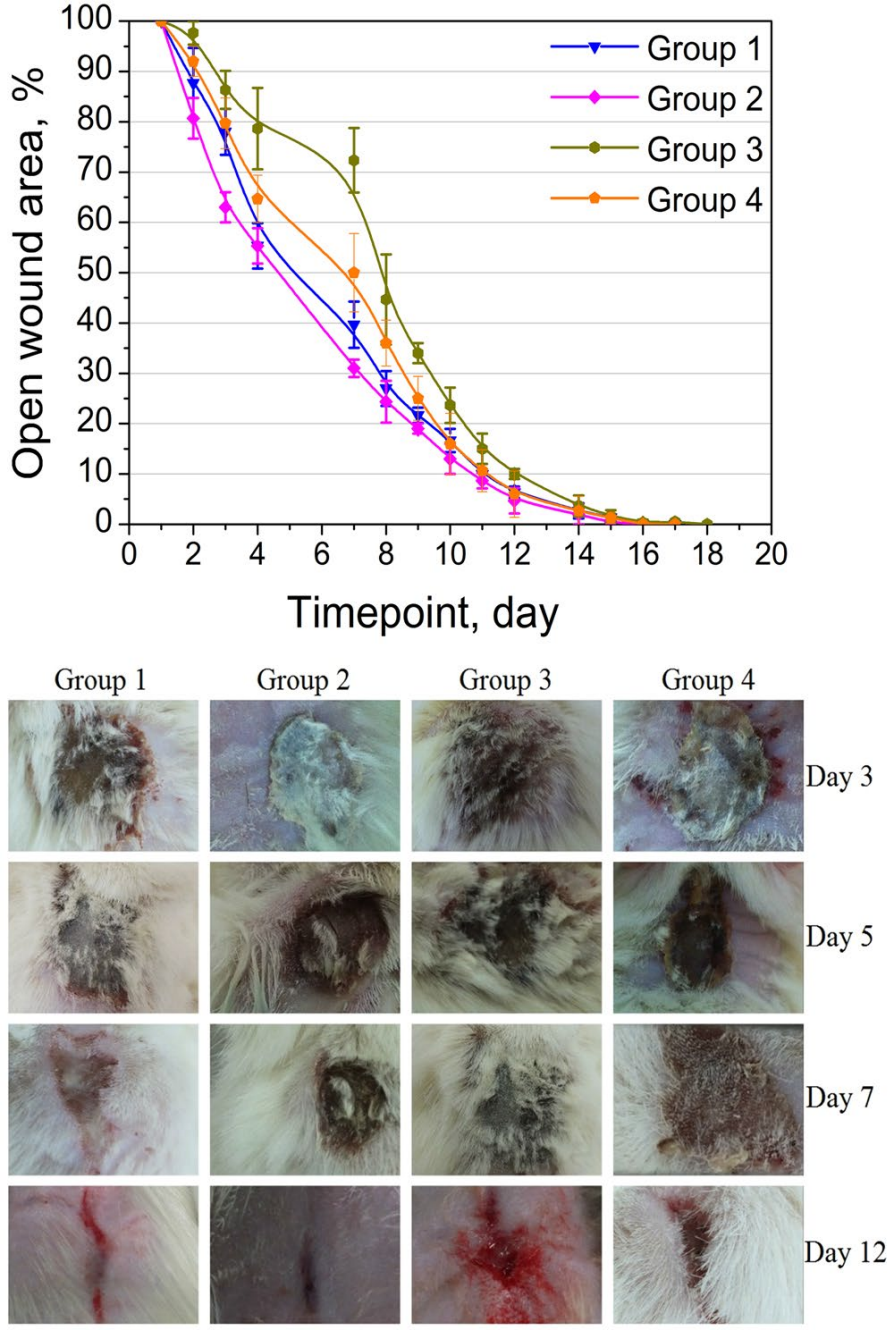
(**A**) Wound surface reduction: Group 1 – healing of untreated 2^nd^ degree burns, Group 2 – second degree burns treated with nano titania, Group 3 – untreated 4^th^ degree burns, Group 4–4^th^ degree burns treated with nano titania. Each value is an average of 3 rats/group (for details, please, see Supplementary Table. S2). All animals survived until the end of the experiment (day 19). (**B**) General appearance of the wounds through the healing process for one representative animal from each group.

In the view of the observed strong effects on blood coagulation potentially attractive for wound healing, it was decided to evaluate the use of titania sol in a spray application on burn wounds *in vivo* in rats that were treated with a pre-heated copper disc, causing burns of second (Groups 1 and 2, untreated and treated respectively) and fourth (Groups 3 and 4, untreated and treated respectively) degree. The rats with untreated wounds were used as controls for both types of incurred damages. Following the healing processes it was possible to note that while the duration of the healing processes in total did not differ appreciably, the dynamics of wound surface reduction was clearly and for more severe wounds even dramatically different. The treatment with a titania sol was apparently resulting in quicker decrease of the exposed wound area, reaching for 4^th^ degree burns as much as 30% reduction in the middle of the healing period (see Fig. 5A,B).

**Figure 6:**
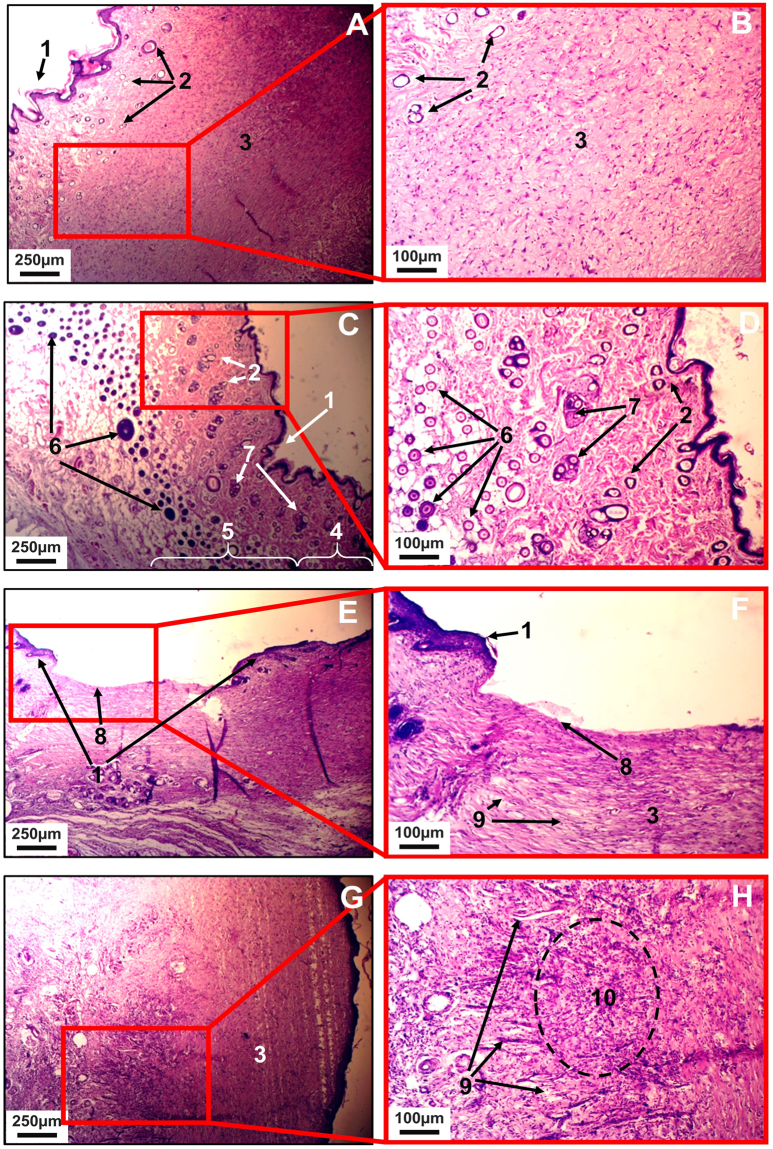
The optical images of healed skin samples, Group 1, second degree burns, non-treated, – (**A**) and (**B**) (x40 and × 100 magnification respectively), Group 2, second degree burns, treated with 0.1 mL TiO_2_ sol daily – (**C**) and (**D**) (x40 and × 100 magnification respectively), Group 3, fourth degree burns, untreated, – (**E**) and (**F**) (x40 and × 100 magnification respectively), and Group 4, fourth degree burns, treated with 0.1 mL TiO_2_ sol daily, – (**G**) and (**H**) (x40 and × 100 magnification respectively). Numbers are tissue structural elements as follows: 1- epidermis; 2 - sweat glands; 3 – scar; 4 – derma; 5 – hypodermis; 6 -hair follicles; 7 - sebaceous glands; 8 – de-epithelialized scar tissue; 9 – scar vessels; 10 - inflammatory infiltration in the scar.

Application of the titania colloid onto the wounds led also to an apparent reduction of the forming scar tissue (see Fig. 6) and its less abnormal appearance. Histological analysis of the healed wound tissues turned to be fully in line with the visual observations. In case of Group 1, i.e. the healing of untreated wounds, the epidermis was not changed, with normal keratinization. In the papillary layer the fibers were thickened and lied more tightly than normal. The number of glands was greatly reduced, in particular, the sebaceous were reduced in size, the sweat epithelium was flattened (with a reduced height of the cells), and hair follicles were isolated (no more than three in sight). The reticular layer was showing more pronounced fibrosis with the activation of fibroblasts, overproduction of the basic substance, and hypervascular focal perivascular leukocyte infiltration (see Fig. 6A,B)).

Moderate fibrosis and adipose tissue of the hypodermis layer of striated muscle fibers. Educated scar was characteristic for burns of the 2 degree.

Histological analysis of the results from Group 2, i.e. healing of second degree wounds treated daily with a titania colloid, gave very exciting and encouraging results. The healed area demonstrated unchanged normal skin structure without any skin structure alterations (see Fig. 6C,D).

For the Group 3, where 4^th^ degree burns were healing without treatment, the epithelium was thinned, sometimes missing, exposing the dense connective tissue that replaced rarely thinned dermis through the entire thickness. Dermal papilla also were smoothed and skin appendages absent. Vascularization of all layers as compared with the sample of Group 4 was reduced. In the deep were observed poorly developed networks of thin-walled vessels with weak perivascular inflammatory infiltration. More fibrosis hypodermis with complete replacement of the connective tissue layer of fat and muscle fibers could be seen (see Fig. 6E,F). Tripe corresponded to 4^th^ degree burns.

For the Group 4, where the 4^th^ degree burns were treated daily with titania, the epithelium was a thickened prickly layer (acanthosis). Dermal papillae were completely smoothed out (unavailable). Dermal thickness was reduced, fibrosis was more pronounced with increasing abundance of fibers and increase in the number of fibroblasts and the base material. Skin appendages - all glands and hair follicles - were completely absent. The reticular layer was a more developed network of young newly formed blood vessels, arranged vertically. Among them - a moderate diffuse leukocyte infiltration, focal hemosiderin deposits and accumulation of hemosiderophages occurred. Severe fibrosis of hypodermis with almost complete replacement of adipose tissue and muscle fiber atrophy took place. Tripe corresponded to the 3rd degree of the burn (Fig. 6G,H).

**Figure 7:**
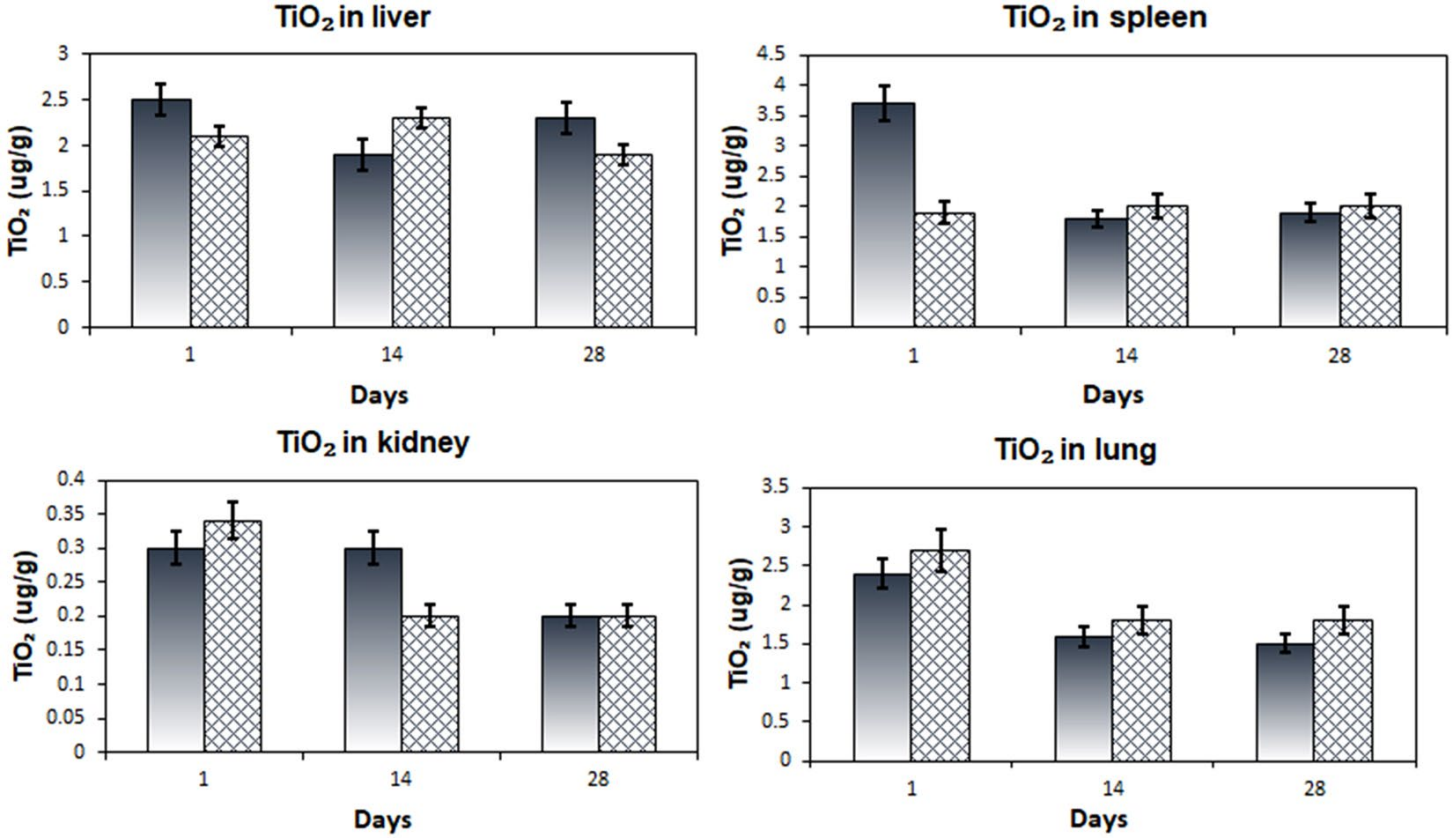
Concentrations of TiO_2_ in the liver, spleen, kidney and lung of the rats untreated, control (to the left), and treated (to the right) by nano dispersion in the course of burn wound healing on 1,14 and 28 days respectively.

The main message of the work presented here is the ability of stabilized anatase TiO_2_ NPs (applied as a solution) to promote burn wound healing tested in an *in vivo* rat model. This was evident by the formation of a firm crust of hybrid protein-titania nanocomposite with significantly higher anti-bacterial and anti-inflammatory properties compared to that of untreated controls. The rats did not reveal any abnormal behavior or apparent pathologies after completion of the healing process. To get an insight into possible side effects in treatment with nano titania we have carried out a thorough investigation of the tissues (liver, kidney, spleen, and brain) of treated animals (and untreated ones as reference) with respect to possible retention of titanium (for details, please, see the description below in Methods). It was clearly demonstrated that the content of titanium did not increase in any of the vital organs of the treated rats, staying at the same level as in the control animals (see Fig. 7 and Supplementary S5 and S6). This result appears quite logical in the view that the applied small TiO_2_ particles possess, as revealed, very strong affinity to proteins. They are apparently either fixed on the surface inside the blood clot or adsorbed directly on the walls if they come into contact with damaged body fluid vessels. It has to be mentioned that the starting level of titanium content in vital organs was in all the studied cases quite low and did not show any statistically appreciable difference between the test and the control samples. The only case, where the difference on the first glance appeared considerable was for the starting spleen samples. It was in this case actually the control that displayed higher titanium content. The reason of the latter might be that the 4 studied rats in this selection (^1^/_3_ of the starting 12 animals sacrificed on the first day of the experiment) have by accident eaten some titanium-containing material (paper, straw or sand). The difference would most probably not be statistically significant if a bigger group of test animals could be investigated, but this would be not ethically acceptable.

Multiple reports are found in the literature describing activities of TiO_2_ immobilized to different matrices, e.g., antibacterial activity *in vitro*^33–35^, as well as anti-inflammatory and accelerated wound healing activity^36^ but to our knowledge, this is the first report of these activities induced by topical application of a solution of TiO_2_ NPs.

Previously, we have studied innate immunity activation by low concentrations of TiO_2_ NPs in whole human blood. We found dose dependent platelet activation, monitored as TAT complex formation, release of thrombospondin-1 (a platelet β-granule protein), and platelet loss. There was substantial activation of the contact/kallikrein system, reflected as generation of FXIIa-AT and FXIIa-C1-INH complexes, and concomitant production and release of the pro-inflammatory chemokines Interleukin (IL-8), Monocyte chemoattractant protein (MCP)-1, Macrophage inflammatory protein (MIP)-1α and MIP-1β (detected using a multiplex analytical panel). All these parameters, except production of MIP-1α and MIP-1β were inhibited by the specific contact system inhibitor CTI^32^. That study confirmed earlier results from our group where we found substantial platelet activation (TAT, platelet loss, release of beta-thromboglobulin [another platelet β-granule protein], generation of FXIIa-AT/ C1-INH, and release of the platelet derived growth factor [PDGF]) induced by planar Ti and TiN surfaces^37^. It should be noted that PDGF was not included in multiplex panel used in^22^ and therefore was not detected in that study.

The link between contact system activation and the release of the same chemokines (IL-8, MCP-1, MIP-1-α, MIP-1β, and PDGF) in addition to vascular endothelial growth factor (VEGF) was also evident in a previous study where we utilized a number of polymers as a tool to investigate these interactions^38^.

In the present work we observe that TiO_2_ NPs greatly accelerates blood clotting *in vitro* in two different models, first by turbidimetry when added to human citrate-plasma, and secondly when evaporated onto polystyrene surfaces which were then incubated with whole human blood. In the latter case, the readouts were TAT generation, platelet loss, and generation of FXIIa-AT and FXIIa-C1-INH complexes, both of which decreased in the presence of CTI.

Cutaneous wound healing is a multistep process where coagulation-induced inflammation is a critical first event^39^. During this initial phase, a protective fibrin clot is formed, platelets are activated to contribute to clot formation, but also to release chemokines and growth factors, which recruit and activate neutrophiles and monocytes. Chemokines, which are essential to promote wound healing include PDGF (chemoattractant for neutrophils, monocytes and fibroblast), IL-8 (the major attract and activator for neutrophils), MCP-1 and MIP-1-α, (which, in conjunction promote macrophage response), MIP-1-β (mixed leukocyte recruiter), and VEGF (which promotes angiogenesis at a late stage in the healing process).

Since the production and secretion of PDGF, IL-8, MCP-1 and MIP-1-α, MIP-1-β, and VEGF all have been shown to be induced by TiO_2_, in conjunction to contact system (FXII) activation, we conclude that this, at least to a certain extent, explains why the administration of TiO_2_ nanopaticles accelerates wound healing.

## Conclusions

Colloidal solution of pH-neutral stabilized titania displayed clear trends to enhanced and accelerated blood clotting. This process was not hindered by addition of common anti-coagulants such as heparin. Interaction of titania dispersion with both blood and skin samples resulted in formation of dense films on the surface with uniform micro cracks caused apparently by contraction of the gel on evaporation of the solvent. The biochemical analysis indicated clearly that this was associated, on one hand, with apparent strong blood clotting ability, and, on the other hand, with activation of the contact system resulting in enhanced wound healing effect. Using the colloidal titania for treatment of burn wounds *in vivo* resulted in apparently quicker reduction of the exposed wound area, while the duration until skin total recovery was comparable with untreated wounds. The most striking effect in application of titania was its logicrent ability to promote restoring of the normal skin structure resulting in the absence of the scar tissue after healing of the 2^nd^ degree burns and improvement of the scar tissue to the appearance typical of a 3^rd^ degree burns in the cases of the 4^th^ degree burn damage.

## Methods

### Preparation of sol-gel titania

The synthesis of the stable size-uniform titania colloids used in this work was made following the earlier described technique^27^. For producing the initial precursor solution Ti(OEt)_4_ (5 mL) was dissolved in anhydrous ethanol (5 mL) and then 1.5 mL of triethanolamine were added on continuous stirring. Hydrolyzing solution (1 mL) was produced by mixing 0.5 M nitric acid, HNO_3_ (0.5 mL), with ethanol, EtOH (2.0 mL). The resulting clear transparent yellowish solution contained 120 mg/mL TiO_2_ according to TGA measurements. The details of particle characterization are provided in the Supplementary.

### Particle characterization

The size of the initial particles in the aqueous sols was measured by dynamic light scattering (Microtrac instrument). FTIR spectra of sols and gels were recorded with a Perkin–Elmer Spectrum 100 instrument without dilution in a cell fitted with CaF_2_ windows. The morphology of the xerogels was studied with a Hitachi TM-1000-μ-DeX 15 kV scanning electron microscope (SEM), and the agglomerate size and crystallinity were studied with a Topcon EM-002 B ultrahigh-resolution analytical electron microscope (TEM). UV/Vis spectra were recorded using a Hitachi U-2001 spectrophotometer.

### Thrombin time test

Lyophilized citrate human plasma and human thrombin (150 NIH units/mg) were obtained from «Kvik» LTD Company, Russia. Thrombin time was measured as a period for clot formation from human citrate plasma with known concentration of plasminogen and fibrinogen. With this aim, 10 mg of lyophilized human plasma was disssolved in 1 mL of triple distilled water (giving final plasminogen concentration-102 μg/mL, fibrinogen concentration – 2.8 mg/mL) and then 0.1 mL of the plasma solution was mixed with 1 mL of 0.9% NaCl solution (isotonic). Thrombin solution was prepared by solving 1 mg of thrombin in 1.5 mL of NaCl solution (isotonic). Clotting mixture was prepared in 1 × 0.5 mm plastic cuvette by mixing 1.1 mL plasma solution and 0.1 mL thrombin solution respectively. Turbidity at 315 nm was immediately monitored during 175 sec. For the tests with titania sol, before addition of thrombin solution 10 or 100 µl titania sol has been added (corresponding to final TiO_2_ content in the mixture of 1 mg/mL and ca. 10 mg/mL respectively) and compared with the samples diluted with the same volumes of isotonic NaCl.

### Blood sampling

Fresh human blood samples were obtained from healthy volunteers who had not received any medication for at least 10 days prior to donation. Blood samples were collected in an open system with no soluble anticoagulant. In this system, any material that comes into contact with blood is furnished with the Corline heparin surface (Corline Systems AB, Uppsala, Sweden) to prevent material-induced contact activation. Preparation followed the manufacturer´s recommendations.

Ethical approval was obtained from the regional ethics committee (Uppsala University Hospital). All methods were carried out in accordance with relevant guidelines and regulations, in particular, complying with the rules summarized by the Swedish Research Council for treatment of human tissue samples summarized at http://www.codex.vr.se/en/manniska4.shtml.

### Skin sampling

The skin samples 1.5–2 mm in diameter were donated by the corresponding author (VGK) and cut by a scalpel from the finger tips.

Written informed consent was obtained from all patients involved in the study.

### The whole blood model

To investigate the influence of the TiO_2_-particles on the blood coagulation cascade in human whole blood a slide chamber model was used, which has been described previously (by Hong *et al*.^40^), containing two circular wells with an inner diameter of 17 mm. The test surfaces with TiO_2_-nanoparticles were prepared by adding 0.5 mL of TiO_2_ particle suspension (120 mg/mL in ethanol) to polystyrene (PS) microscope slides followed by evaporation overnight. As a reference PS slides were treated the same way, but without the TiO_2_-nanoparticles. The chambers, the control surface (PVC) and the tubes, tips and tubing to be used in contact with the blood were pre-coated with heparin (Corline Systems AB). Blood was drawn from healthy volunteers, who not had received any medication at least 10 days prior to blood donation.

The wells were filled with 1.5 mL freshly drawn blood containing 0.5 IU/mL heparin (Leo Pharma) and the test surface was attached with two clips, thereby constituting a lid over the two chamber wells. These devices were then incubated under constant rotation at 30 rpm for 60 min. at 37 °C. After incubation the blood was mixed with EDTA at a final concentration of 10 mM to inhibit further activation of the blood cascade systems. Before centrifugation platelet counts were performed. The blood samples were then centrifuged at 2500 g for 15 min., the plasma was collected and stored at −70 °C for further analysis of coagulation markers. The experiment was repeated four times (different blood donors each time) in duplicates. To one series of experiments 3.5 μM Corn Trypsin Inhibitor (CTI; Enzyme Research Laboratories), which is a specific FXIIa inhibitor, was added to the blood prior to incubation with the surfaces.

### Platelet count

The number of platelets was analyzed in the blood samples before and after incubation with the test surfaces using a Sysmex XP-300 Hematology Analyzer (Sysmex Corp.). Platelet count was calculated as the remaining amount as compared to the initial sample (before incubation in the chambers) and was expressed as mean percent of initial ± SEM.

### Thrombin-Antithrombin complexes (TAT) ELISA

Plasma levels of TAT were analyzed by a conventional sandwich ELISA. The plasma samples were diluted in normal citrate-phosphate-dextrose plasma. The TAT complexes were captured by an anti-human thrombin antibody (Enzyme Research Laboratories) and detected with an HRP-conjugated anti-human AT antibody (Enzyme Research Laboratories). As standard pooled human serum diluted in in normal citrate-phosphate-dextrose plasma was used. All values were given in μg/L.

### Contact activation complexes

For the detection of FXIIa-antithrombin (AT) and FXIIa-C1-inhibitor (C1INH) complexes in the plasma samples a standard sandwich ELISA described by Sanchez *et al*.^41^ was used. Microtiter plates were coated with anti-human FXIIa antibodies (Enzyme Research Laboratories) and captured complexes were subsequently detected with either biotinylated anti-human AT (Dako) or biotinylated anti-human C1INH (Enzyme Research Laboratories) followed by HRP-conjugated streptavidin (GE Healthcare). Standard solutions were diluted in normal plasma. All measured values are given in nmol/L.

### *In vivo* Investigation of burn wound healing properties

Male Hooded rats (body weight range 200–250 g) were used for the study. Animals were acclimatized under standard animal laboratory condition for 7 days prior to the experiment. All experiments were approved by institutional animal ethical committee (Ivanovo State Medical Academy, Russia, Protocol No. 2 from 06.04.2015) and are in agreement with the guidelines for the proper use of animals for biomedical research^42^. Animals were divided into 4 groups, each consisting of 3 rats: I group – rats with 10 sec treatment with heated disc; II group – rats with 10 sec treatment with heated disc and healing titania; III group – rats with 20 sec treatment with heated disc; IV group – rats with 20 sec treatment with heated disc and healing titania. All animals survived and did not suffer weight loss within standard deviation until the last day of the experiment (day 19).

Animals were anesthetized with ketamine (dose 60 mg/kg), acting as both sedative and long-term pain-killer agent^43^, by intraperitoneal injection, the dorsal hair was shaved and disinfected. Burns were made 1 cm diameter copper disc preliminary heated up to 300 °C. For groups II and IV the materials were applied on excised burns. The burns were treated daily with 0.1 mL of prepared titania solution. Wound sizes were measured daily until the healing is complete. The wound outline was transferred to transparent films and scanned with an Epson Perfection 2480 scanner. The wound area was calculated with ImageJ 1.30 v. software. The percentage wound reduction was calculated according to the following formula:

1 $${C}_{n}=[({S}_{0}\mbox{--}{S}_{{\rm{n}}})/{S}_{0}]\,\times \,100$$

where C_n_ is the percentage of wound size reduction, S_o_ is initial wound size, S_n_ is wound size on respective day.

The rats were kept in individual cages 20 × 30 cm^2^ area and had free ability to motion and access to both food and water. As the wounds were located in the dorsal area, there was no risk that the animals should lick or bite their own wounds.

### Histological analysis

Fragments of skin with scar excised and completely fixed in 10% formalin solution during 24 hours. After routine gynecological wiring samples were poured into paraffin. 20 slices with 5 μm thickness were prepared and stained with hematoxylin and eosin from each paraffin block.

### Chemical analysis of tissue samples

Analysis of the content of titania in the organs was carried out according the following procedure. Animals (in the experiment, 12 rats were used) on 1, 14 and 28 days respectively after wound healing with titania were anesthetized with isoflurane and were killed by cervical dislocation and organs (liver, kidney, spleen and lung) were collected and weighed immediately after killing of the animals. Dissolution of organs was carried out with a mixture of concentrated sulfuric and nitric acids. Completeness of dissolution was achieved by organs heating in heat-resistant glasses. 1 ml of concentrated HNO_3_ and 3 ml of concentrated HCl were added to the resulting syrup-like transparent solutions with following transferring to 25 ml volumetric flasks using distilled water. The titanium concentration was determined by atomic-absorption spectroscopy with inductively coupled plasma. The studies were carried out on HORIBA Jobin Yvon ULTIMA 2. Three rats without titania treatment were used as a control sample for organs.

The *in vivo* experiment was repeated for another group of animals and in this case liver, and brain tissues were removed from one representative animal per group, cut into pieces about 0,2 g that were weighed and then dissolved in 3 ml of aqua regia. The pH after dissolution was adjusted to 3.0 by addition of 1.0 M NH_3_ solution. The produced liquids were analyzed with ICP-AES Spectro Cirros CCD Instrument, Kleve, Germany.

Two of the authors, GAS and VGK declare their involvement in the activities of the CaptiGel AB company, Sweden, developing metal oxide colloids for environmental and biomedical applications.

## Electronic supplementary material

Supplementary information
