# Author Correction: Dispersion of TiO_2_ nanoparticles improves burn wound healing and tissue regeneration through specific interaction with blood serum proteins

**DOI:** 10.1038/s41598-018-22717-8

**Published:** 2018-03-08

**Authors:** Gulaim A. Seisenbaeva, Karin Fromell, Vasiliy V. Vinogradov, Aleksey N. Terekhov, Andrey V. Pakhomov, Bo Nilsson, Kristina Nilsson Ekdahl, Vladimir V. Vinogradov, Vadim G. Kessler

**Affiliations:** 1 Department of Chemistry and Biotechnology, BioCenter, Swedish University of Agricultural Sciences, Box 7015, SE-750 07 Uppsala, Sweden; 2 Department of Immunology, Genetics and Pathology, Rudbeck Laboratory C5:3, Uppsala University, SE-751 85 Uppsala, Sweden; 3 Laboratory of Solution Chemistry of Advanced Materials and Technologies, ITMO University, Kronverksky Pr. 49, St, Petersburg, 197101 Russian Federation; 4 Ivanovo State Medical Academy, Sheremetevskiy prosp. 8, Ivanovo, 153012 Russian Federation; 5 Linnæus Centre for Biomaterials Chemistry, Linnæus University, SE-391 82 Kalmar, Sweden

Correction to: *Scientific Reports* 10.1038/s41598-017-15792-w, published online 13 November 2017

The Acknowledgements section in this Article is incomplete.

“The authors are indebted to Prof. Nicholas A. Kotov at the Michigan University for fruitful discussions and recommendations in the preparation of the manuscript. This work was supported by the Russian Government, Ministry of Education (research was made possible due to financing provided to the customer from the federal budget aimed at maximizing teh customer’s competitive advantage among the world’s leading educational centers) and by the RFBR, Research Project Nos.14-03-31046 and 14-03-00502. The support from the Swedish Research Council (Vetenskapsrådet), Grant No. 2014-3938, is gratefully acknowledged.”

should read:

“The authors are indebted to Prof. Nicholas A. Kotov at the Michigan University for fruitful discussions and recommendations in the preparation of the manuscript. This work was supported by the Russian Government, Ministry of Education (research was made possible due to financing provided to the customer from the federal budget aimed at maximizing teh customer’s competitive advantage among the world’s leading educational centers) and by the RFBR, Research Project Nos.14-03-31046 and 14-03-00502. The support from the Swedish Research Council (Vetenskapsrådet), Grant No. 2014-3938, is gratefully acknowledged. Vladimir V. Vinogradov acknowledges the Ministry of Education and Science of Russian Federation (Project 4.8955.2017/8.9).”

