# Supplementary material for: Dispersion of TiO2 nanoparticles improves burn wound healing and tissue regeneration through specific interaction with blood serum proteins
Source: Sci Rep. 2017 Nov 13;7:15448. doi: 10.1038/s41598-017-15792-w (PMC5684224; doi:10.1038/s41598-017-15792-w)
Supplement: Supplementary file 1 — Supplementary information [file 41598_2017_15792_MOESM1_ESM.pdf]

Supplementary materials

Dispersion of TiO_2 nanoparticles improves burn wound healing and tissue regeneration through specific interaction with blood serum proteins

Gulaim A. Seisenbaeva,[1] Karin Fromell,[2] Vasiliy V. Vinogradov,[3] Aleksey N. Terekhov,[4] Andrey V. Pakhomov,[4] Bo Nilsson,[2] Kristina Nilsson Ekdahl,[2,5] Vladimir V. Vinogradov,[3] Vadim G. Kessler,[1]*

1 Department of Chemistry and Biotechnology, BioCenter, Swedish University of Agricultural Sciences, Box 7015, SE-750 07 Uppsala, Sweden,

2Department of Immunology, Genetics and Pathology, Rudbeck Laboratory C5:3, Uppsala University, SE-751 85 Uppsala, Sweden,

3 Laboratory of Solution Chemistry of Advanced Materials and Technologies, ITMO University, St. Petersburg, 197101, Russian Federation,

4 Ivanovo State Medical Academy, Ivanovo, 153012, Russian Federation

5 Linnæus Centre for Biomaterials Chemistry, Linnæus University, SE-391 82 Kalmar, Sweden,

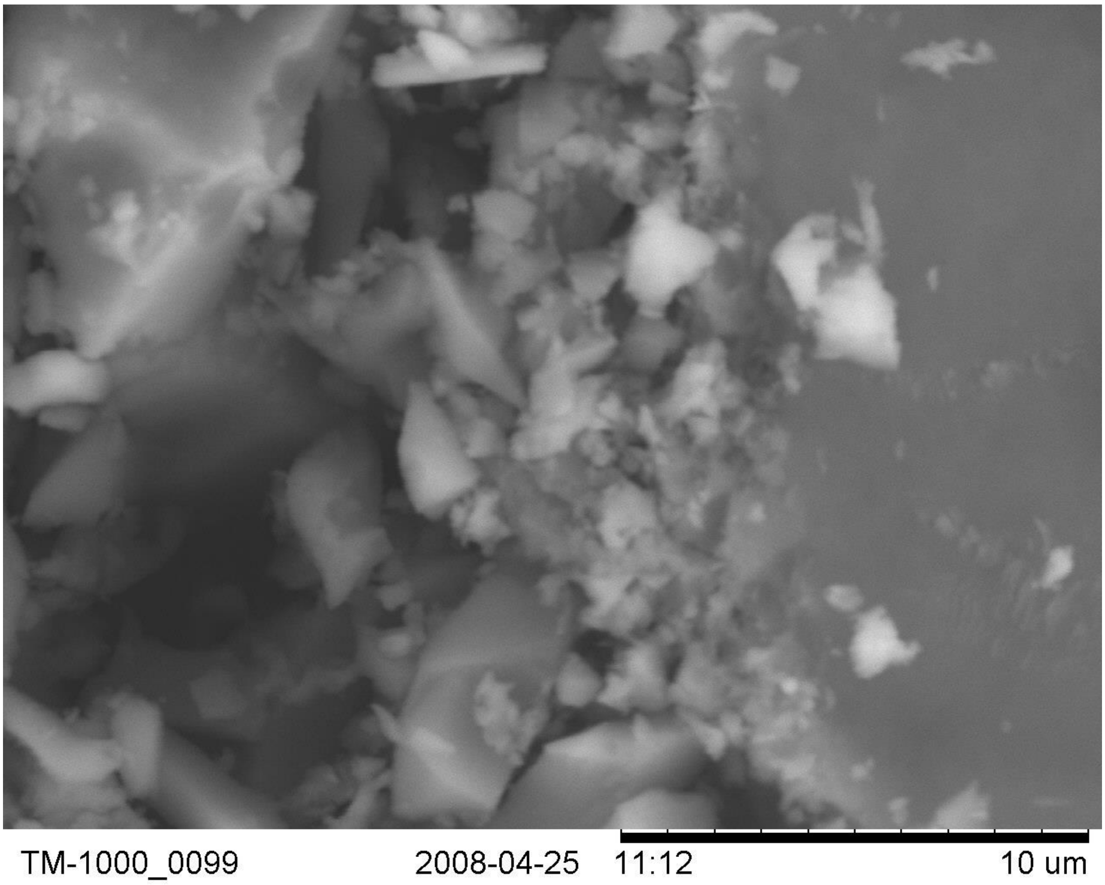

Figure FS1. SEM view of the vacuum dried original TiO_2 sol diluted by isotonic NaCl solution 1:1 by volume.

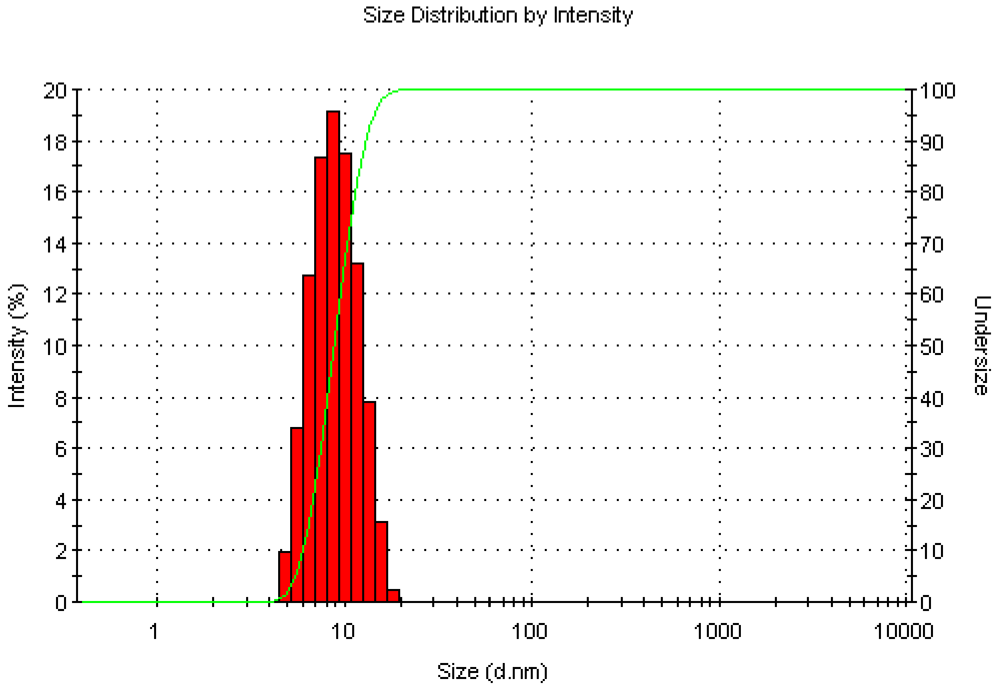

Figure FS2 Size distribution of the TiO_2 nanoparticles in the sol diluted by isotonic NaCl solution 1:1 by volume.

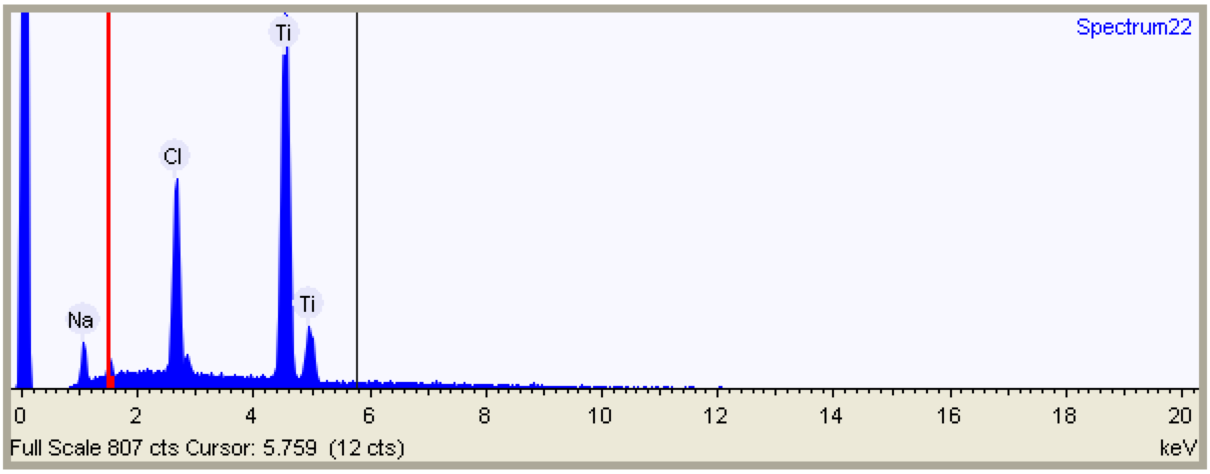

Figure FS3. EDS spectrum of the vacuum dried original TiO_2 sol diluted by isotonic NaCl solution 1:1 by volume.

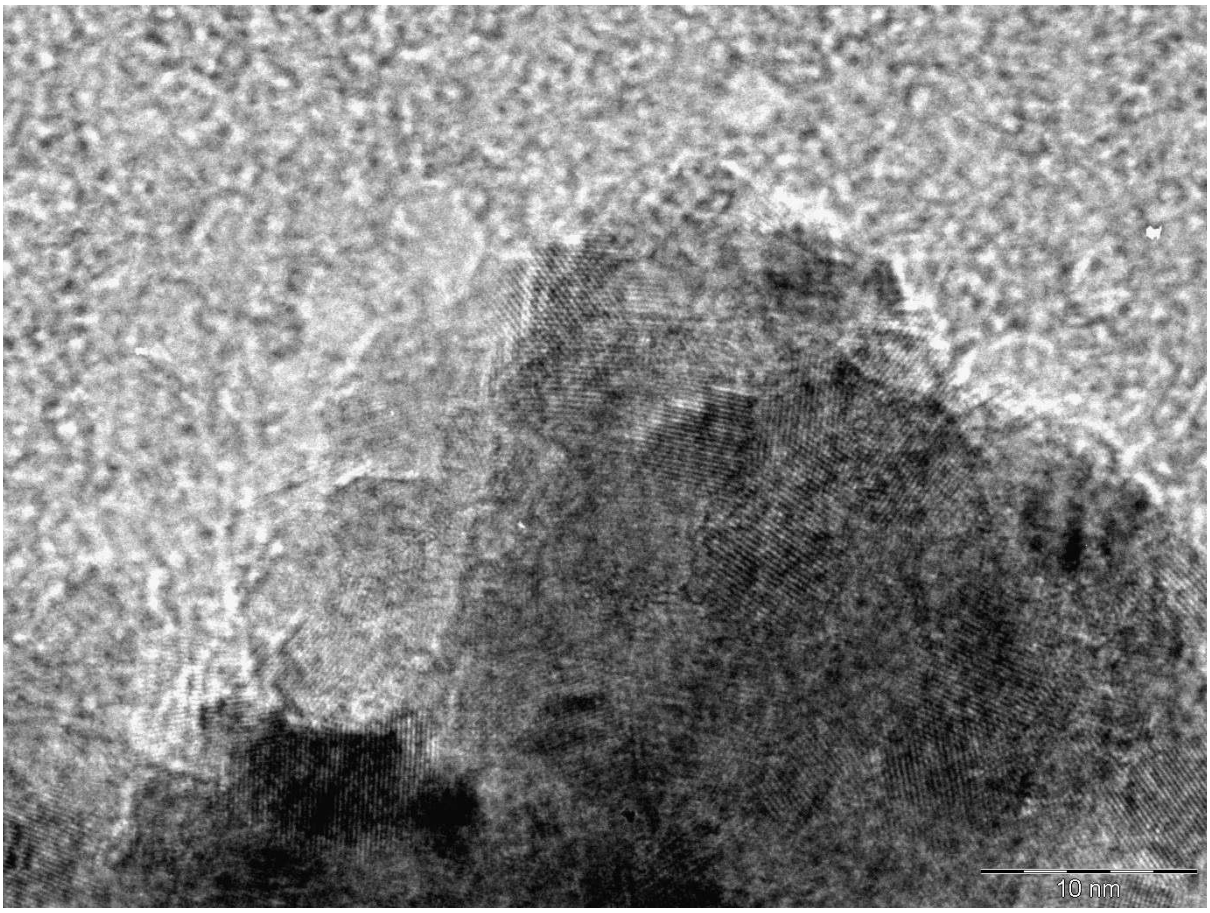

Figure FS4. TEM view of the TiO_2 nanopartices in an air-dried droplet of the sol.

Table TS1 Results of EDS analysis with normalized content of all identified elements

TiO_2 coating on a blood clot

Element	Weight %
Chlorine	0.9
Potassium	1.0
Titanium	98.1

Inner part of the TiO_2 coated blood clot

Element	Weight %
Sodium	1.9
Magnesium	6.1
Phosphorus	13.3
Sulfur	44.7
Potassium	20.6
Calcium	2.2
Titanium	2.5
Iron	8.6

Table TS2 Wound size development in the % of the initial size.

Group 1

Day	Animal 1	Animal 2	Animal 3
1	100	100	100
2	95	81	87
3	82	73	79
4	60	55	51
7	45	37	37
8	31	25	25
9	23	20	22
10	18	18	14
11	10	10	10
12	5	7	7
14	1	3	4
15	1	1	2
16	0	0	0
17	0	0	0
18	0	0	0
19	0	0	0

Group 2

Day	Animal 4	Animal 5	Animal 6
1	100	100	100
2	85	77	80
3	66	60	63
4	59	52	55
7	30	30	33
8	23	29	21
9	18	19	20
10	16	13	10
11	7	10	9
12	2	5	7
14	0	2	4
15	0	0	1
16	0	0	0
17	0	0	0
18	0	0	0
19	0	0	0

Group 3

Day	Animal 7	Animal 8	Animal 9
1	100	100	100
2	99	95	99
3	89	82	88
4	86	80	70
7	77	75	65
8	55	39	40
9	36	32	34
10	27	20	24
11	15	12	18
12	11	9	10
14	6	2	3
15	3	1	1
16	1	0	0
17	0,5	0	0
18	0	0	0
19	0	0	0

Group 4

day	Animal 10	Animal 11	Animal 12
1	100	100	100
2	95	89	92
3	85	79	75
4	70	61	63
7	55	41	54
8	40	31	37
9	23	22	30
10	20	9	19
11	12	6	14
12	5	2	11
14	1	1	6
15	0,5	0	2
16	0	0	0
17	0	0	0
18	0	0	0
19	0	0	0

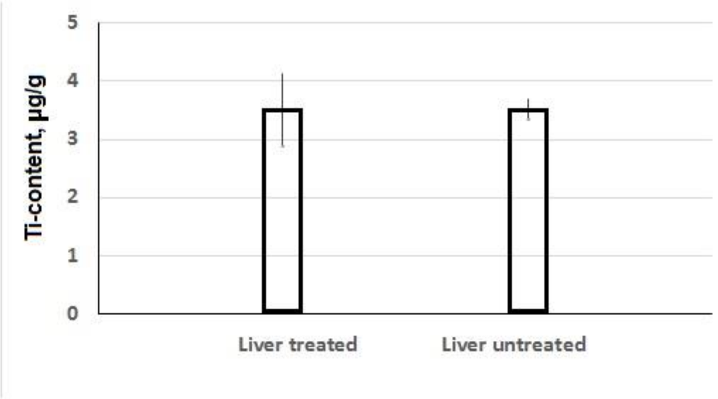

Figure FS5. Concentrations of TiO_2 in the liver of the rats treated and untreated (control) by nano dispersion measured on the day 28 from the start in an repeated experiment

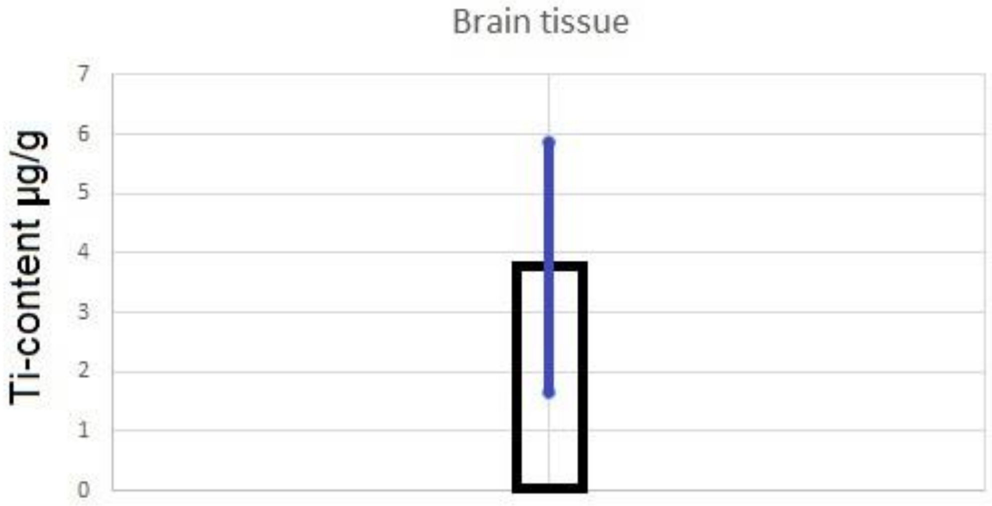

Figure FS6. Concentrations of TiO_2 in the brain tissue of the rats treated with titania. The observed value of 3.77±2.11 μg/g is well corresponding to that generally observed in rats in this kind of tissue (see, for example, I.Grissa et al. *Environ Sci Pollut Res*, **2016**, *23*, 20205‑20213; X.Y. Gao, *Biol Trace Elem Res*, **2011**, *143*, 1616–1628; H.B. Shi et al. Particle & Fibre Toxicol, **2013**, *10*, 15 (1-33). doi: 10.1186/1743-8977-10-15).
